# Out-of-hospital cardiac arrest in dialysis patients

**DOI:** 10.1007/s11255-020-02694-6

**Published:** 2020-12-18

**Authors:** Marta Obremska, Katarzyna Madziarska, Dorota Zyśko, Jerzy R. Ładny, Robert Gałązkowski, Mariusz Gąsior, Klaudiusz Nadolny

**Affiliations:** 1 Department of Preclinical Research, Wroclaw Medical University, Wroclaw, Poland; 2 Department and Clinic of Nephrology and Transplantation Medicine, Wroclaw Medical University, Borowska St. 213, 50-556 Wroclaw, Poland; 3 Department of Emergency Medicine, Wroclaw Medical University, Wroclaw, Poland; 4 Department of Emergency Medicine, Medical University of Bialystok, Bialystok, Poland; 5 Department of Emergency Medical Service, Medical University of Warsaw, Warsaw, Poland; 6 Department of Cardiology, Silesian Center for Heart Diseases, Faculty of Medical Sciences in Zabrze, Medical University of Silesia, Katowice, Poland; 7 Department of Emergency Medical Service, Higher School of Strategic Planning in Dabrowa Gornicza, Dabrowa Gornicza, Poland; 8 Faculty of Medicine, Katowice School of Technology, Katowice, Poland

**Keywords:** Out-of-hospital cardiac arrest, Dialysis, Cardiopulmonary resuscitation

## Abstract

**Purpose:**

The aim of the study was to assess whether a history of dialysis is related to cardiopulmonary resuscitation (CPR) attempts and survival to hospital admission in patients with out-of-hospital cardiac arrest (OHCA).

**Methods:**

The databases of the POL-OHCA registry and of emergency medical calls in the Command Support System of the State of Emergency Medicine (CSS) were searched to identify patients with OHCA and a history of dialysis. A total of 264 dialysis patient with OHCA were found: 126 were dead on arrival of emergency medical services (EMS), and 138 had OHCA with CPR attempts. Data from the POL-OHCA registry for patients with CPR attempts, including age, sex, place of residence, first recorded rhythm, defibrillation during CPR, and priority dispatch codes, were collected and compared between patients with and without dialysis.

**Results:**

CPR attempts by EMS were undertaken in 138 dialyzed patients (52.3%). The analysis of POL-OHCA data revealed no differences in age, sex, place of residence, first recorded rhythm, and priority dispatch codes between patients with and without dialysis. Defibrillation was less frequent in dialysis patients (*P* = 0.04). A stepwise logistic regression analysis revealed no association between survival to hospital admission and a history of hemodialysis (odds ratio = 1.12; 95% CI 0.74–1.70, *P* = 0.60).

**Conclusions:**

A history of dialysis in patients with OHCA does not affect the rate of CPR attempts by EMS or a short-term outcome in comparison with patients without dialysis. Defibrillation during CPR is less common in patients on dialysis than in those without.

## Introduction

The number of people receiving dialysis in Poland reaches 540 per million inhabitants and has been growing by about 1.3% per year, but it is still lower than the average number reported for other European Union countries (710 per million) [1]. Cardiac arrest accounts for a quarter of deaths among patients on dialysis [2, 3]. It is known that early initiation of cardiopulmonary resuscitation (CPR) is associated with better survival and quality of life in patients with cardiac arrest [4].

Although all-cause mortality rates in dialysis patients are constantly decreasing, the frequency of sudden cardiac death (SCD) remains stable [5]. The annual risk of SCD is higher in patients on dialysis than in general population, patients not receiving dialysis in end-stage renal disease (ESRD), or even patients with heart failure [6]. Studies on cardiac arrest in dialysis patients concern the incidence of cardiac arrest during hemodialysis session, which may be provided at a dialysis center located in or out of hospital [7–9]. It is known that rapid electrolyte and fluid shifts during dialysis may cause electrical instability and predispose to cardiac arrhythmia, especially in the presence of left ventricular hypertrophy (LVH) or reduced ejection fraction [10, 11]. Additionally, the progression of cardiovascular diseases in dialysis patients is affected not only by traditional risk factors but also by other factors associated with uremia [12, 13]. Ventricular arrhythmias are more likely to cause sudden cardiac arrest (SCA) during hemodialysis sessions [14, 15]. Studies based on electrocardiogram recordings have shown that in the nondialysis period, SCA is more often caused by bradycardia and subsequent asystole [16, 17].

There are scarce data on the prevalence and treatment of out-of-hospital cardiac arrest (OHCA) in dialysis patients [18]. These patients constitute a minority of the whole population of patients with OHCA and are poorly represented in small studies on OHCA. A history of dialysis is one of the factors that decrease the rates of survival to hospital discharge in patients admitted after CPR due to OHCA [19]. Although comorbidities worsen prognosis in OHCA patients, the importance of hemodialysis as a factor related to the incidence of CPR attempts and survival to hospital admission has not been investigated so far. Therefore, the aim of this study was to assess the associations between dialysis status and the rate of CPR attempts and survival to hospital admission in patients with OHCA.

## Methods

### Patient population and data source

#### Hemodialysis in Poland

According to a national registry from 2018, dialysis therapy in Poland was conducted in 275 dialysis centers, including 131 public (48%) and 142 private (52%) ones. However, data on the number of dialysis patients in 2018 are not fully consistent due to considerable fluctuations in the status of patients as a result of transplant procedures, moving to a different dialysis center, or death.

Patients who have cardiac arrest at an in-hospital dialysis center (usually a public one) are managed in the hospital and are considered as having in-hospital cardiac arrest (IHCA). On the other hand, if patients suffer cardiac arrest at a private dialysis center, they immediately receive medical assistance on the spot and the emergency medical services (EMS) are called.

### Study setting

**Fig. 1:**
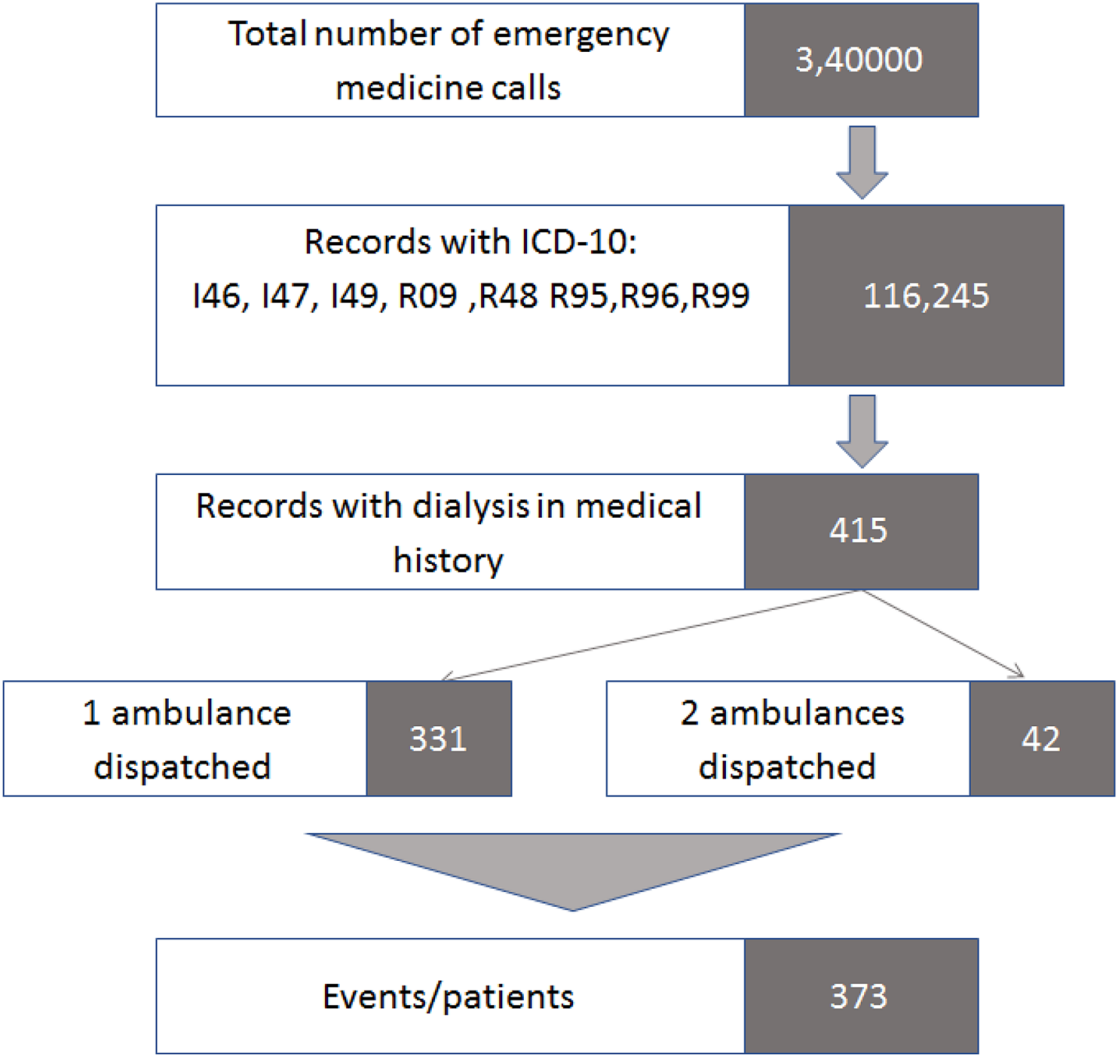
Flow chart of patients/event inclusion from Command Support System of the State Emergency Medicine

The study was designed as a retrospective analysis based on the 2018 POL-OHCA (Poland OHCA) registry of OHCA and a database of emergency medical calls in the Command Support System of the State of Emergency Medicine (CSS) [20]. Because POL-OHCA contains only cases of cardiac arrest with CPR attempts, the database of the emergency medical calls from CSS was searched to identify dialysis patients with OHCA and those who pronounced dead on arrival of EMS. We searched for the International Classification of Diseases, 10th Revision codes that were likely to be used in patients with OHCA, including I46, I47, and I49 as well as R09, R95, R96, R98, and R99. A total of 116,245 records from all 340,000 in 2018 were retrieved, which were then searched using the term “dializa” (dialysis) in the field “medical history”. This yielded 415 records related to 373 events (2 ambulances were dispatched to 42 events) (Fig. 1). Data regarding the history of dialysis were based only on information reported by the caller. Most cardiac arrests occur at home, which implies that there usually is a person who knows the patient’s history. Moreover, callers generally report that the patient is on dialysis as they consider this to be important information. For the purpose of this study, the terms “dialysis” and “hemodialysis” were used interchangeably, because “dialysis” was used to retrieve information from the CSS database. The rate of peritoneal dialysis in Poland is only 5% of dialysis patients; therefore, we assume that it does not affect the obtained results.

The status (dead or alive) of a patient after EMS treatment is not clearly stated in the database of the CSS. Therefore, it was assessed on the basis of records related to medical treatment, vital signs, and information whether the patient was referred to the hospital or remained at the scene of emergency.

**Fig. 2:**
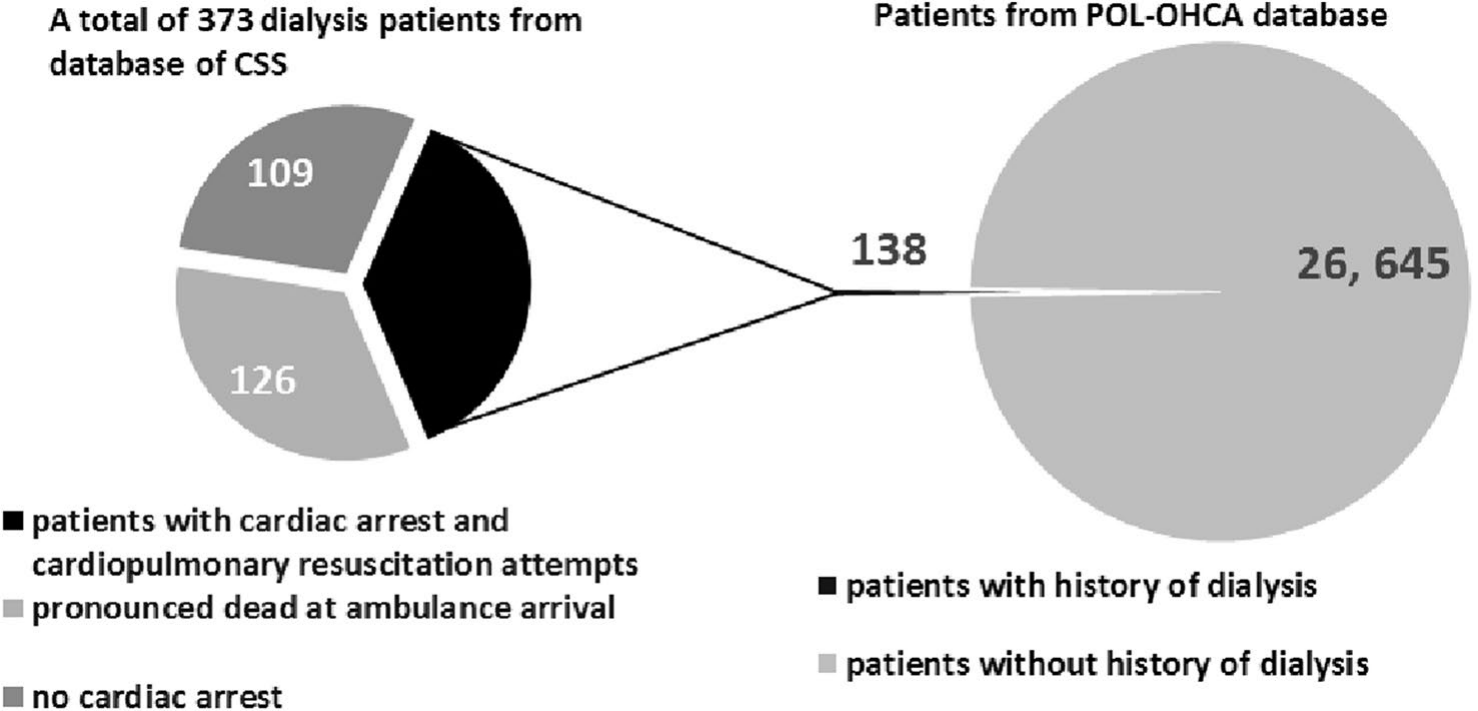
Distribution of dialysis patients in database of Command Support System of the State Emergency Medicine and in Pol-OHCA registry

Among the 373 patients, 109 had no cardiac arrest, 126 were dead on arrival of EMS, and 138 patients had cardiac arrest and underwent CPR (defibrillation or at least one dose of epinephrine). The selected records from the CSS database were reviewed and combined with information obtained from the POL-OHCA registry, which included data of 26,783 patients with CPR attempts due to OHCA in 2018 in Poland (Fig. 2). The following data were collected for the 138 dialysis patients included in the POL-OHCA database and compared between individuals with CPR attempts with and without a history of dialysis recorded by an emergency dispatcher: age, sex, place of residence, first recorded rhythm, CPR attempt, defibrillation during CPR, and priority dispatch codes (code 1, which denotes the highest priority and requires the use of visual and audible signaling by the ambulance, or code 2, which does not require signaling unless the dispatcher decides otherwise, for example, due to traffic congestion).

### Ethics approval

As this was a retrospective study, the approval of the Ethics Committee and patient consent to participate in the study were not required.

### Statistical analysis

Continuous variables were presented as means and standard deviations and compared with the Student’s *t* test. Discrete variables were presented as numbers and percentages and compared with the chi-squared test. A stepwise logistic regression analysis was conducted to find an association between survival to helicopter EMS (HEMS) transport or hospital admission and demographic and clinical data. The “dialysis” variable was retained in the stepwise logistic regression analysis even if *P* was not significant. A *P* value of less than 0.05 was considered significant.

## Results

The final study sample included 264 patients with OHCA and a history of dialysis, including 138 patients who underwent CPR (epinephrine and/or defibrillation) and 126 patients who were pronounced dead on arrival of EMS. Therefore, CPR attempts were undertaken in at least 52.3% of dialysis patients with OHCA. Among the 138 patients with CPR attempts, resuscitation efforts were unsuccessful in 87 patients and 51 patients survived to hospital admission or HEMS transport. A comparison between OHCA patients with and without a history of dialysis who underwent CPR and were included in the POL-OHCA database is presented in Table 1.

**Table 1:** Comparison of patients with out-of-hospital cardiac arrest with and without a medical history of dialysis who underwent cardiopulmonary resuscitation

Parameter	History of dialysis (*N* = 138)	No history of dialysis (*N* = 26,645)	*P* value
Age (years), mean ± SD	67.1 ± 11.8 (*N* = 127)	65.6 ± 17.6 (*N* = 22,638)	0.33
Male sex	107 (67.3)	19,930 (68.4)	0.83
Defibrillation	33 (23.9)	8536 (32.0)	0.04
Place of residence (city > 10,000 inhabitants)	77 (55.8)	14,644 (55.0)	0.84
Priority code 1	118 (86.8)	23,325 (90.2)	0.36
2 EMS ambulances dispatched	21 (15.2)	4256 (16.0)	0.81
Defibrillation performed by the first EMS	32 (23.2)	8370 (31.4)	0.04
VF/VT recognized by the first EMS	22 (16.4)	5448 (20.8)	0.22
Sinus rhythm	19 (13.8)	2475 (9.3)	0.07
Supraventricular tachycardia	1(0.7)	374 (1.4)	0.50
Ventricular tachycardia	1 (0.7)	227 (0.9)	0.87
Atrial fibrillation	2 (1.5)	1026 (3.9)	0.14
Asystole	91 (65.9)	18,057 (67.8)	0.65
PEA	30 (21.7)	6641 (24.9)	0.39
Pacemaker artifacts	6 (4.4)	510 (1.9)	0.04
ECG signs of myocardial infarction	2 (1.5)	849 (3.2)	0.25
Pacing	4 (2.9)	462 (1.7)	0.30
Intubation	109 (79.0)	20,632 (77.4)	0.66
Venous access	126 (91.3)	24,719 (92.8)	0.51
Traumatic injuries	6 (4.4)	3182 (11.9)	0.006
Epinephrine	135 (97.8)	25,665 (96.3)	0.35
Atropine	23 (16.7)	5424 (20.4)	0.28
Survival to hospital admission or HEMS transport	51 (36.9)	9682 (36.3)	0.88

Data are presented as number (percentage) of patients unless otherwise indicated

*VF/VT* ventricular fibrillation/ventricular tachycardia, *PEA* pulseless electrical activity, *EMS* emergency medical services, *HEMS* helicopter emergency medical service

The rate of OHCA on Mondays and Tuesdays was 42.0% in patients on dialysis and 29.1% in those not undergoing dialysis (*P* < 0.001).

**Fig. 3:**
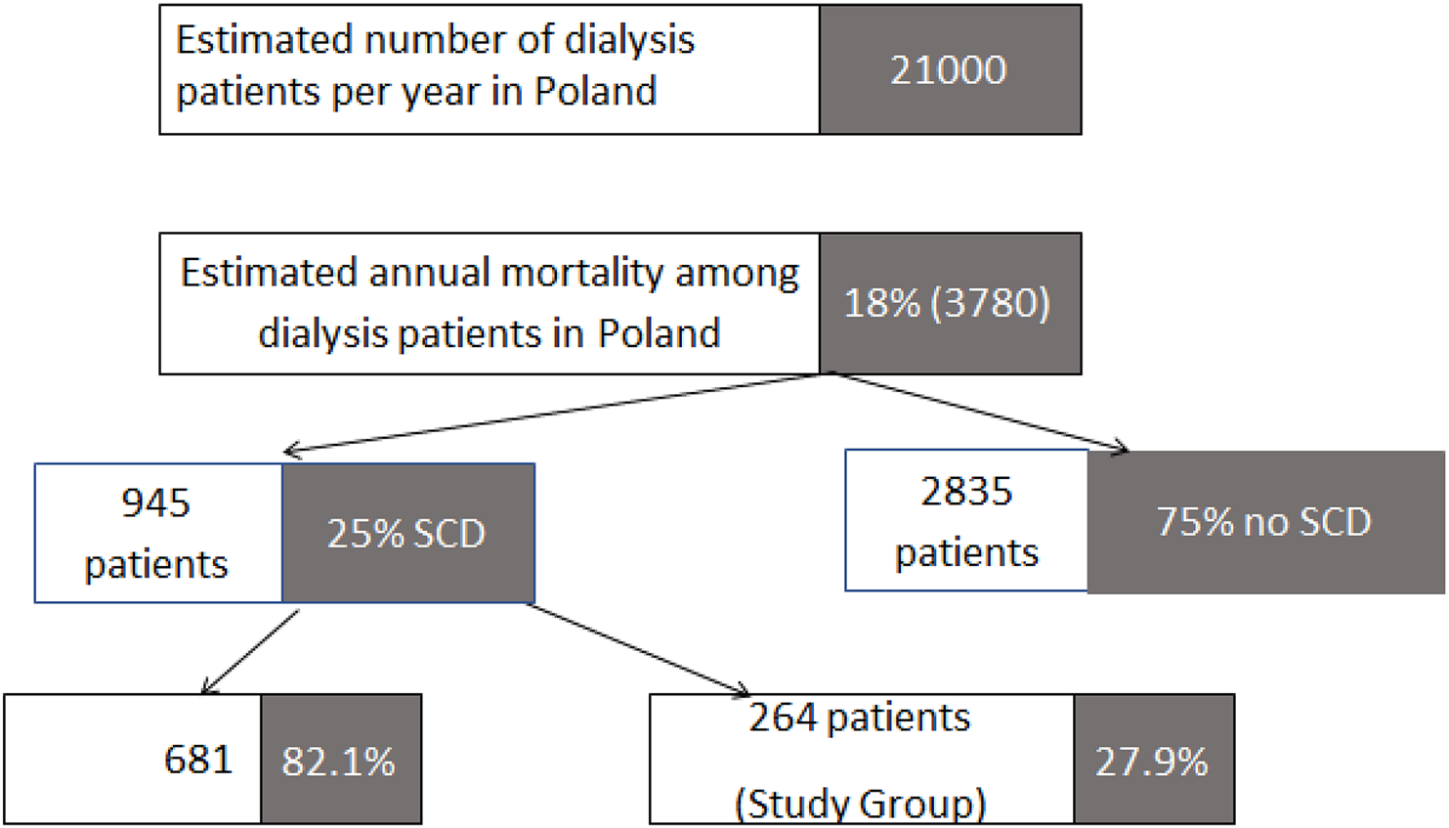
Total annual rates of sudden cardiac death in dialysis patients

In dialysis patients, the rate of ventricular fibrillation (VF) or pulseless ventricular tachycardia (VT) on Mondays and Tuesdays was 13.8%, but on the other days of the week, it was 17.5% (*P* = 0.59). In nondialysis patients, the rate of VF/VT on Mondays and Tuesdays was 21.1%, as compared with 20.2% on the other days (*P* = 0.01). The rate of VF/VT was similar on Mondays and Tuesdays (*P* = 0.17) as well as on the other days of the week (*P* = 0.55) in patients with and without a history of dialysis (Fig. 3).

### Multivariate analysis

In the stepwise logistic regression analysis, the following factors were related to survival to hospital admission or HEMS transport: VT or VT as the first recorded rhythm, defibrillation, atropine use, place of residence (city of more than 10,000 inhabitants), and priority code 2. On the other hand, male sex and epinephrine use were linked to worse survival (Table 2).

**Table 2:** Association between survival to hospital admission or helicopter emergency medical services transport and demographic and clinical data: a stepwise logistic regression analysis

Parameter	OR	95% CI	*P* value
VF or VT on first ECG	1.73	1.57–1.90	< 0.001
Defibrillation	1.29	1.18–1.40	< 0.001
Atropine use	1.16	1.08–1.25	< 0.001
Place of residence (city > 10,000 inhabitants)	1.21	1.14–1.29	< 0.001
Priority code 2	1.82	1.66–1.99	< 0.001
Male sex	0.86	0.80–0.91	< 0.001
Epinephrine use	0.18	1.15–1.22	< 0.001
Hemodialysis	1.12	0.74–1.70	0.60

*ECG* electrocardiogram, *OR* odds ratio, *VF* ventricular fibrillation, *VT* ventricular tachycardia

## Discussion

Our study revealed that more than half of patients with a history of dialysis underwent CPR by EMS. This rate is similar to that reported for the general population of patients with OHCA in previous research [21]. This finding indicates that the history of dialysis does not affect CPR attempts undertaken by EMS personnel.

The second finding of our study is that a history of dialysis is not related to survival to hospital admission or HEMS transport, either in a univariate and multivariate analysis. Shockable first recorded rhythm and VF during CPR, as well as defibrillation performed during CPR, are well-known positive predictors of survival [22]. Surprisingly, despite the lack of differences in survival, defibrillation was performed less often in dialysis patients with OHCA than in those not receiving dialysis. The lower rate of defibrillation in dialysis patients is expected to be related with worse survival. However, it could not be excluded that hyperkalemia in dialysis patients, a frequent cause of OHCA, can be successfully treated during CPR. Sacher et al. showed that higher potassium levels are associated with a greater risk of conduction disturbances [17]. Dialysis patients are at risk of high potassium levels, especially after a longer break in dialysis. Electrolyte disturbances in these patients may be responsible for the lack of benefit from an implantable cardioverter-defibrillator (ICD) implantation. Pun et al. did not show any difference in mortality in dialysis patients depending on the ICD implantation in primary prevention, which is consistent with the results of the MADID II study in a subgroup of patients with low glomerular filtration rate [23, 24]. Wase et al. showed a progressive increase in the defibrillation threshold with increasing renal failure, particularly in patients with progression of LVH [25]. About 60–80% of patients starting dialysis have LVH [26]. Its regression is associated with improved survival in ESRD patients [27]. Additionally, considering the periprocedural complications associated with ICD implantation in dialysis patients, the procedure requires an individualized approach in this population [28].

Our study also revealed that OHCA is more frequent on Mondays and Tuesdays than on the other days of the week, which is in line with a study by Kiuchi et al. [10]. Dialysis according to the Mon–Wed–Fri or Tue–Thu–Sat schedule is preferable from the logistic point of view; however, to reduce the rate of cardiac arrest, the daily schedule seems to be more advantageous [29].

A study among dialysis patients showed that patients with CPR undertaken during dialysis by medical personnel had three-fold higher survival rates compared with dialysis patients in whom CPR was performed by EMS [9]. However, the benefits of using automated external defibrillator were not demonstrated because only 37% of patients presented a shockable rhythm. Nevertheless, the study emphasized the importance of a rapid initiation of CPR by bystanders also in the group of dialysis patients, which is in line with our study.

Dialysis patients were reported to have a worse outcome of in-hospital cardiac arrest than those not receiving dialysis [30]. This is contrast to a recent study conducted using the Get With The Guidelines-Resuscitation registry, which showed that the outcome of dialysis patients is similar to those not receiving dialysis [31]. However, it is unclear to what extent the method adopted in the study allows to distinguish dialysis patients from the whole population.

The frequency of SCD per year could be estimated using data from other studies. The annual mortality rate among dialysis patients in Poland was reported to reach 18% [1]. Based on the CRUSH-ILS study, sudden death occurs in a quarter of hemodialysis patients [3]. Therefore, assuming that the estimated annual number of dialysis patients is 21,000, the annual number of deaths would be 3780, including 945 SCAs. Our population of OHCA patients receiving dialysis included 264 patients, that is, approx. 30% of the entire expected population of dialysis patients with SCD. Therefore, the population seems to be sufficiently representative to draw conclusions. The SCA in dialysis patients occurs more frequent than in general population, which indicates the need to look for predictive factors and possible modifications of CPR in renal failure [32].

The major limitation of our study is its retrospective design and the inherent problem of incomplete medical records. Another limitation is the fact that some OHCA patients with CPR attempts and no reported history of dialysis might have actually received dialysis. However, we estimate that there were only about 2% of such patients, which is unlikely to have affected the results. We were also unable to assess whether OHCA in our patients was sudden. However, this is a common problem in large epidemiologic studies where detailed medical history is unavailable. Therefore, the term “OHCA” is preferred over SCA in these studies.

## Conclusions

In conclusion, a history of dialysis is not a factor that discourages EMS teams to perform CPR and affect the rate of CPR attempts. Moreover, a history of dialysis is not related to worse short-term outcome in dialysis patients in comparison with the general OHCA population. Finally, defibrillation during CPR is less likely to be performed in patients receiving dialysis.
